# A dataset for the flood vulnerability assessment of the upper Cross River basin using morphometric analysis

**DOI:** 10.1016/j.dib.2020.105344

**Published:** 2020-02-28

**Authors:** Nkpa Ogarekpe, Ekpe Obio, Imokhai Tenebe, PraiseGod Emenike, Chidozie Nnaji

**Affiliations:** a Department of Civil Engineering, Cross River University of Technology, Calabar, Nigeria; b Department of Agronomy, Cross River University of Technology, Calabar, Nigeria; c Ingram School of Engineering, Texas State University, San Marcos, TX, USA; d Department of Civil Engineering, Covenant University, Ota, Nigeria; e Department of Civil Engineering, University of Nigeria, Nsukka, Nigeria

**Keywords:** Flood, Vulnerability, Upper cross river basin, Morphometric analysis

## Abstract

The on-site collection of data is not only time consuming, but expensive and perhaps near impossible in restive communities within the upper Cross River basin (UCRB). Therefore, the importance of this data cannot be overemphasized. This article presents a Digital Elevation Model (DEM), land use and land cover (LULC) map, soil map, geology map and climatic datasets which enhance the understanding of the physical characteristics of the upper Cross River basin using morphometric analysis. The use of the LULC map, soil map and the DEM in conjunction with the climatic data enhance the creation of the Hydrologic Response Units (HRUs) and the water balance modelling. The simulation of the water balance at the HRU level enables the routing of the runoff to the reaches of the sub-basins and then to the channels. The geology map provides confirmatory information to the morphometric analysis. The compound factor computed from all the derived morphometric parameters enhance the determination of the overall flood potential of the congruent sub-basins.

**Table:** Specifications Table

Subject	Environmental Science (General)
Specific subject area	Flood vulnerability assessment
Type of data	Table Images
How data were acquired	The datasets were obtained from the British Geological Survey, Food and Agricultural Organization of the United Nations (FAO-UNESCO), WaterBase and the National Centers for Environmental Prediction (NCEP), Texas, USA, for free.
Data format	The raw and analyzed datasets are made available in the following formats TIFF, SHP, TXT, XLSX.
Parameters for data collection	The choice of data and the conditions for data collection were made based on the research need and the recommendations of previous researches.
Description of data collection	The DEM was obtained from the Shuttle Radar Topographic Mission (http://srtm.csi.cgiar.org/) The land use and land cover map was obtained from the WaterBase website (http://www.waterbase.org/download_data.html.) The soil map was collected from the Land and Water Development Division, Food and Agricultural Organization of the United Nations website http://www.fao.org/soils-portal/soil-survey/soil-maps-and-databases/en/The climatic data and Weather Generator were obtained from the National Centers for Environmental Prediction (NCEP), Texas, USA (http://globalweather.tamu.edu/) The geology map was obtained from British Geological Survey https://www.bgs.ac.uk/africagroundwateratlas/downloadGIS.html
Data source location	The collected data are for the area between longitudes 7°33′ and 10°06′ East and latitudes 4°55′ and 7°26′ North
Data accessibility	data.mendeley.com/datasets/rz42cbstpb/draft?a=c405043e-f9e3-45e5-af88-ed7f2b034b79

**Table:** 

**Value of the Data** • These datasets provide a comprehensive understanding of the physical characteristics of the upper Cross River basin considering the morphometry of the congruent sub-basins. • Considering their roles to providing useful information of the basin characteristics, the data can be used by policy makers for the effective mitigation and management of the flood vulnerabilities of the upper Cross River basin. • The datasets are also useful for the modelling of the impact of land use and land cover change on the hydrology of the UCRB, and the impact of climate change on the water balance of the basin.

## 1 Data description

**Fig. 1:**
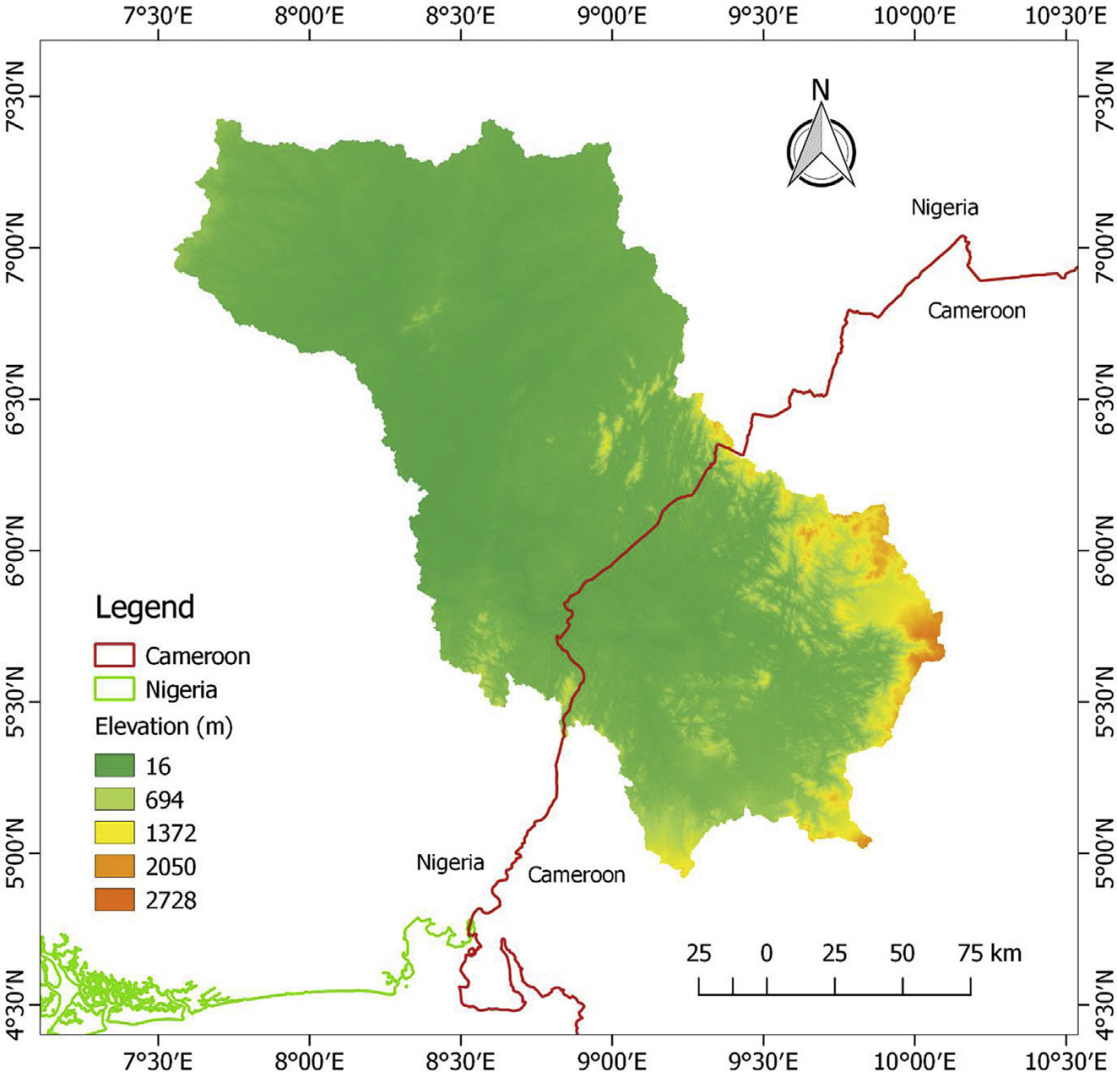
DEM of upper Cross River basin located in Southeastern Nigeria and Western Cameroon.

**Fig. 2:**
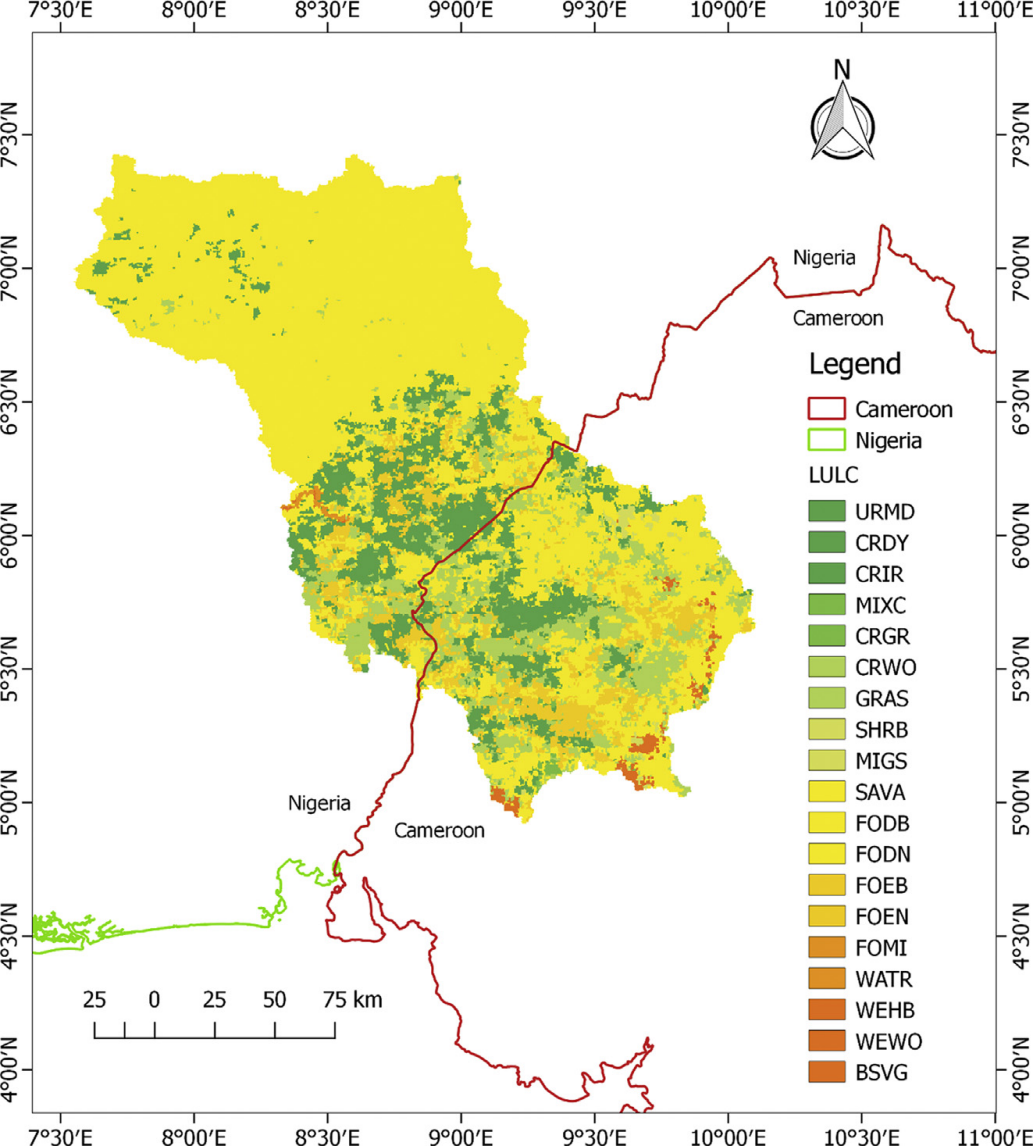
Land use and land cover map of upper Cross River basin.

**Fig. 3:**
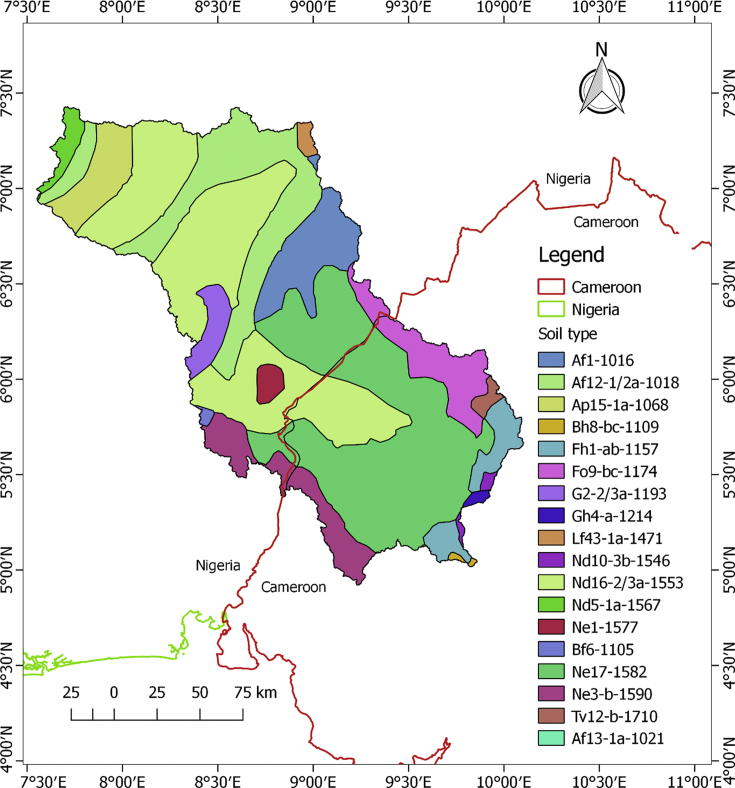
Upper Cross River basin soil map.

**Fig. 4:**
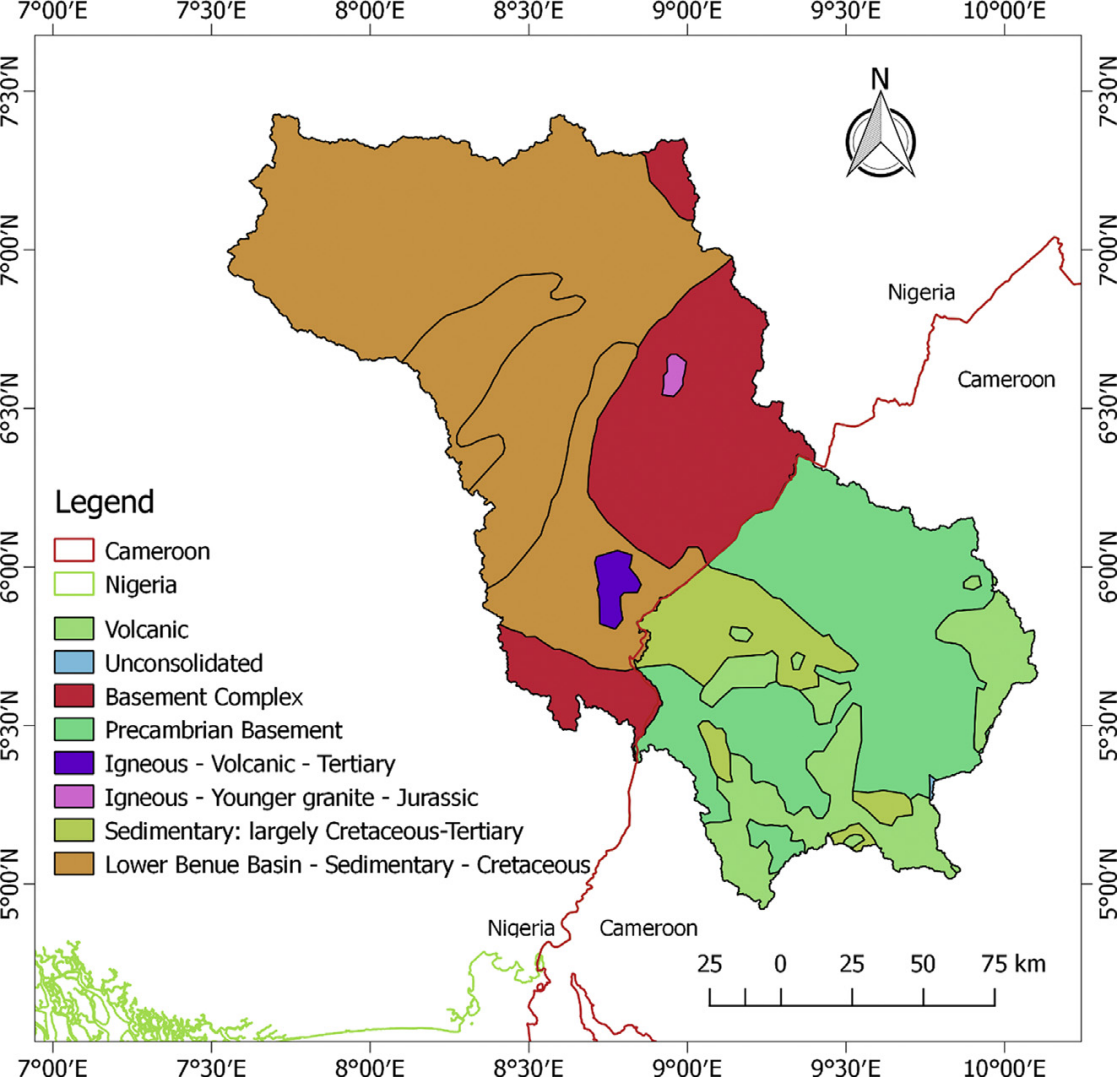
Upper Cross River basin geology map.

The datasets in this article describe the land use and land cover, soil, geology, topography and the climatic condition of the upper Cross River basin. The delineation of the watershed offered by the Soil and Water Assessment Tool (SWAT) model was used to clip out the datasets. This was considered necessary in order to lay emphasis on the area under review. Fig. 1 describes the topography of the upper Cross River basin located in Southeastern Nigeria and Western Cameroon. Fig. 2 displays the land use and land cover of the watershed while Fig. 3 depicts the soil types within the watershed. The lithological formations of the upper Cross River basin are shown in Fig. 4. The climatic data consist of precipitation, minimum/maximum temperature, wind speed, relative humidity, and solar radiation datasets.

## 2 Experimental design, materials, and methods

### 2.1 Study area

**Fig. 5:**
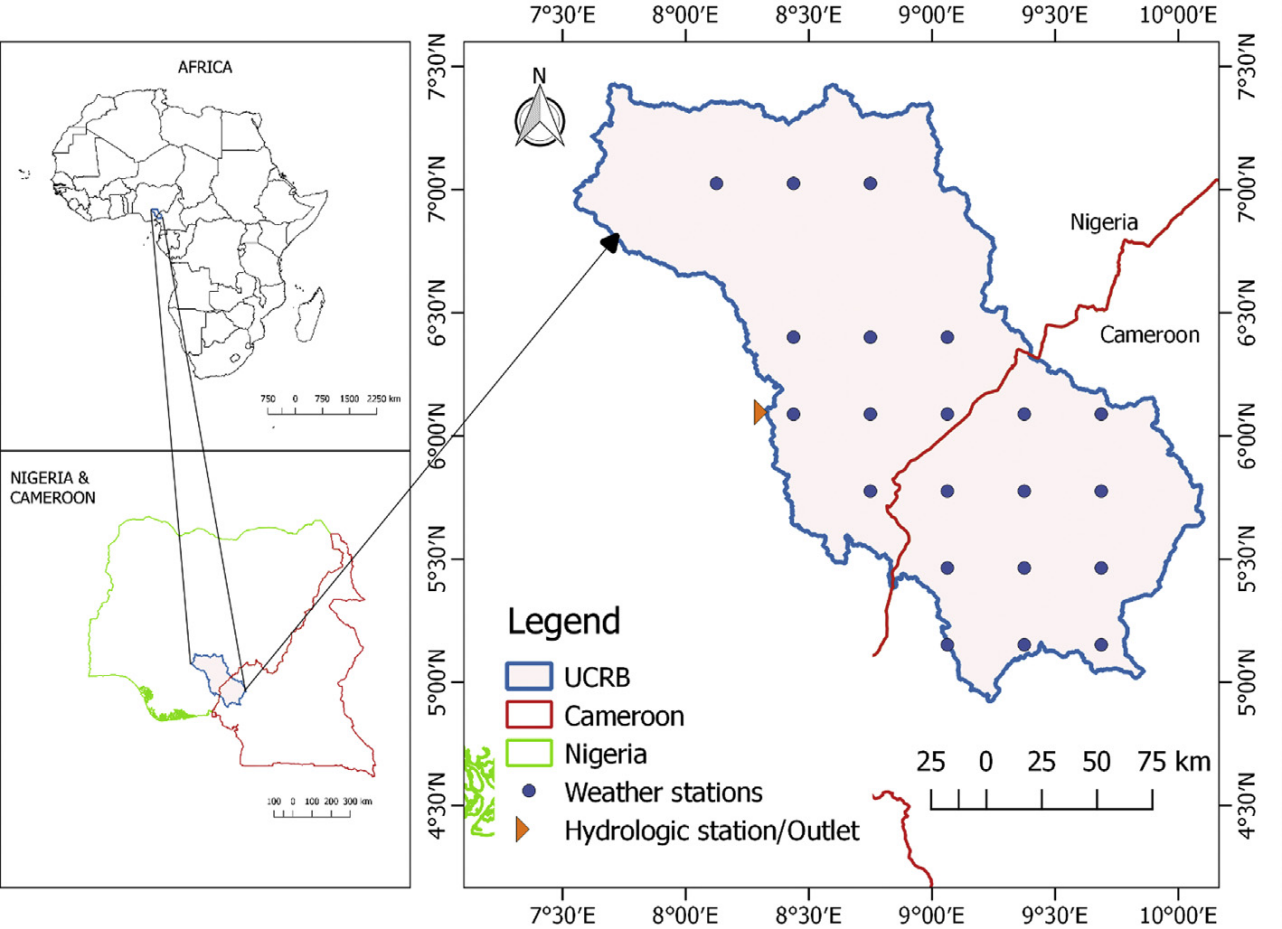
Upper Cross River basin located in Southeastern Nigeria and Western Cameroon shown with weather stations.

The upper Cross River basin (UCRB) is located in Southeastern Nigeria and Western Cameroon. Cross River originates from Cameroon, flows through Nigeria and drains into the Atlantic Ocean. The upper Cross River basin lies within Nigeria and Cameroon, between longitudes 7°33′ and 10°06′ East and latitudes 4°55′ and 7°26′ North. The UCRB has an area of 35,942.84 km^2^. The watershed experiences a tropical wet-and-dry climate based on Koppen's climate classification. The location map is shown in Fig. 5.

The variability of the elevations of the UCRB is shown in Fig. 1. The UCRB consist of a nexus of low-lying and mountainous areas, ranging from 16 to 2728 m above mean sea level. Fig. 1 further shows that most of these low-lying areas are in Nigeria while the mountains are situated in Cameroon.

The distribution of the LULC extracted from Fig. 2 shows that the predominant LULC by percentage of watershed are as follows: savanna (SAVA 63.36%), Dryland Cropland and Pasture (CRDY 14.33%), Cropland/Woodland Mosaic (CRWO 9.95%) and Evergreen Broadleaf Forest (FOEB 8.22%). The other land uses/land cover by percentages of watershed are Urban and Built-up Land (URMD 0.02%), Cropland/Grassland Mosaic (CRGR 0.36%), Grassland (GRAS 1.20%), Shrubland (SHRB 0.52%), Deciduous Broadleaf Forest (FODB 0.95%), Water Bodies (WATR 0.21%), Wooded Wetland (WEWO 0.82%) and Barren or Sparse Vegetated (BSVG 0.07%).

Fig. 3 details the different soil types within the UCRB. The soil types are described based on the Food and Agricultural Organization of the United Nation (FAO-UNESCO) classifications. Table 1 gives the compositions of the soil by percentage of the watershed under review.

**Table 1:** Composition of upper Cross River basin soil by percentage of watershed.

Soil type symbol	Percentage composition of watershed (%)
Af1-1016	6.43
Af12-1-2a-1018	13.84
Af13-1a-1021	0.03
Ap15-1a-1068	4.51
Bf6-1105	0.12
Bh8-bc-1109	0.30
Fh1-ab-1157	2.45
Fo9-bc-1174	5.51
Gh4-a-1214	0.69
G2-2-3a-1193	0.64
Lf43-1a-1471	0.55
Nd5-1a-1567	0.56
Nd10-3b-1546	0.55
Nd16-2-3 a-1553	28.75
Ne1-1577	0.79
Ne3-b-1590	5.52
Ne17-1582	28.10
Tv12-b-1710	0.66

The predominant soil types are the Dystric Nitosols, Eutric Nitosols and Ferric Acrisols. Acrisols and Nitosols have significant amount of clay. The Dystric Nitosols within the UCRB consist of medium to fine texture particles, with gentle undulating relief.

The predominant lithological formations of the UCRB are sedimentary and basement rocks (Fig. 4). The varieties of the formations by percentage of watershed are as follows: volcanic (9.03%), basement complex (17.08%), igneous-volcanic (0.75%), igneous – younger granite (0.24), Precambrian basement (22.28%), sedimentary: Cretaceous – tertiary (5.88%), Lower Benue Basin - Sedimentary – Cretaceous (44.71%) and unconsolidated (0.03%).

Climatic data such as daily precipitation, minimum/maximum temperature, wind speed, relative humidity and solar radiation, were obtained for a period of 36 years, spanning the time frame between 1979 and 2014. The weather station (WGEN) file of the UCRB was created using the Weather Generator program and the daily climatic data as input. The Weather Generator program enables the simulation of missing weather stations statistics required by QSWAT for a complete cycle of modelling. The climatic data reveal that the UCRB has an average annual rainfall of 3049.5 mm.

### 2.2 Model input data source

The data were remotely obtained from different sources. The Digital Elevation Model (DEM) of the UCRB was obtained from the Shuttle Radar Topographic Mission (http://srtm.csi.cgiar.org/). The 90 m resolution DEM was used for the watershed delineation. The delineation was carried out considering an outlet at 8.25°E and 6.05°N (Hydrologic station). All the spatial analyses were carried out using QGIS (versions 2.6) set to WGS 84/UTM Zone 32 N Coordinate Reference System. The LULC map was obtained from the WaterBase website (http://www.waterbase.org/download_data.html) while the soil map was obtained from the Land and Water Development Division, Food and Agricultural Organization of the United Nations website http://www.fao.org/soils-portal/soil-survey/soil-maps-and-databases/en/. The climatic data was obtained from the National Centers for Environmental Prediction (NCEP), Texas, USA (http://globalweather.tamu.edu/). Also, the Weather Generator program was obtained from the Soil and Water Assessment Tool website (http://globalweather.tamu.edu/).

### 2.3 SWAT model

QSWAT installed as a plugin in QGIS is required for the geoprocessing operations. The geoprocessing operations are performed by QSWAT via a suite of programs called Terrain Analysis Using Digital Elevation Models [1,2]. QSWAT being a plugin, utilizes QGIS tools and functions including the Geospatial Data Abstraction Library (GDAL). The SWAT model utilizes the LULC map, soil map and DEM in conjunction with the climatic data in the creation of the Hydrologic Response Units (HRUs) and the water balance modelling. The water balance of the catchment area simulated at the HRU level enables the routing of the runoff to the reaches of the sub-basins and then to the channels.

### 2.4 Model setup

The discretization of the congruent sub-basins that makeup the UCRB can be carried out using a threshold area of 10 km^2^. The definition of slope classes is important for the HRU creation step. The specification of four slope classes such as: 0–5%, 5–10%, 10–25% and >25% is not out of order. The multiple HRU option of filtering by land use, soil and slope can be used for the creation of HRU. HRUs with less than 5% unique combination of land use, soil and slope range can be eliminated to reduce the model complexity, processing and simulation time.

### 2.5 Morphometric analysis

TauDEM of QSWAT1.2 enables the terrain analysis and the determination of the morphometric parameters. The drainage, flow direction, flow accumulation, slope, aspect, hill shade and the morphometric parameters are generated consequent upon the model input data, SWAT model and the model setup described in 2.2, 2.3, 2.4 above. The requisite morphometric parameters include the stream length (Lu), stream number (Nu), stream order (Su), area of the sub-basins (A), minimum (h) and maximum (H) elevations. The use of the geospatial tools in QGIS aid the determination of the sub-basin length (L_b_) and perimeter (P). The derived parameters can therefore be obtained using the mathematical expressions shown in Table 2.

**Table 2:** Morphometric parameters and computational procedure.

S/NO	Morphometric parameters	Computation procedure	Reference
1	Stream Order (S_u_)	Hierarchical	Strahler [3]
2	Stream Number (N_u_)	N_u_ = N_1_ + N_2_ + … +N_n_	Schumn [4]
3	Stream Length (L_u_)	L_u_ = L_1_ + L_2_ + … +L_n_	Horton [5]
4	Bifurcation Ratio (R_b_)	R_b_ = N_u_/(N_u__+1_)	Schumn [4]
5	Form Factor (F)	R_f_ = A/L_b_^2^	Horton [6]
6	Circularity Ratio (C)	C = 4πA/P^2^	Miller [7]
7	Compactness Index (c)	Ci = 0.2821 P/√A	Horton [6]
8	Drainage Density (D_d_)	D_d_ = L_u_/A	Horton [6]
9	Drainage Texture (T_d_)	Dt = ∑N_n_/P	Horton [5]
10	Elongation Ratio (E_b_)	E_b_ = [√(A/π) ]/L_b_	Schumm [4]
11	Stream Frequency (F_s_)	F_s_ = ∑N_n_/A	Horton [6]
12	Lemniscate ratio (K)	L_b_^2^/4A	Chorley et al. [8]
13	Basin relief (H_r_)	H_r_ = H - h	Hardley and Schumm [9]
14	Relief ratio (R_r_)	Rr = H_r_/L_b_	Schumm [4]
15	Ruggedness number (R_n_)	Rn = H_r_ x D_d_	Melton [10]

Where N_u_ is the total number of stream of a given order, N_u__+1_ is the total number of stream of next higher order, L_u_ is the total stream length of all orders (km), A is area of the sub-basin (Km^2^), L_b_ is the maximum basin length (Km), P is the perimeter of the basin (Km), D_d_ is the drainage density (Km/Km^2^).

### 2.6 Implications of the data

The geology, soil, topography and LULC of the watershed aid the understanding of the physical characteristics of the watershed. In respect of the influence of the sub-basins on the flooding of the main channel of the UCRB, the most flood vulnerable areas are underlain by hard rocks with high relief, hence, greater runoff, low permeability and infiltration capacity. A sparsely vegetated land, with impermeable surface and high relief, has the potential to attain peak discharge in a short period of time. Consequently, the instantaneous high runoff contribution of their tributaries to the main channel. The moderate-to-low flood vulnerable areas are low lying areas, underlain by porous formations, characterized with high permeability and infiltration capacity with lower runoff. The assessment of the flood vulnerability of watersheds is based on the understanding of the nexus of the various characteristics of the watershed.

In order to determine the flood vulnerabilities of the sub-basins, the derived parameters in respect of the linear, aerial and relief parameters are weighted on a scale of 1–18. The choice of the range of weightages is dependent on the number of sub-basins within the basin. Higher weightages are assigned to sub-basins with higher derived parameter values. Hence, sub-basins with the highest derived parameter values are assigned a weightage of 18, while sub-basins with the second highest derived parameter values are assigned a weightage of 17 and so on. The sub-basins with the least derived parameter values are assigned a weightage of 1. The compound factors of the sub-basins are calculated by determining the averages of the weightages of all the derived parameters. Sub-basins with high compound factors are of high priority while those with low compound factors are of low priority.
